# Inulin supplementation modulates gut microbiota derived metabolites related to brain function in children with obesity

**DOI:** 10.1038/s41598-025-21079-2

**Published:** 2025-10-07

**Authors:** Tushar Andriyas, Sira Sriswasdi, Rossarin Tansawat, Jaraspong Uaariyapanichkul, Sirinuch Chomtho, Chonnikant Visuthranukul

**Affiliations:** 1 Department of Food and Pharmaceutical Chemistry, Faculty of Pharmaceutical Sciences, Chulalongkorn University, Bangkok, 10330 Thailand; 2 Center of Excellence in Metabolomics for Life Sciences, Chulalongkorn University, Bangkok, 10330 Thailand; 3 Research Affairs, Faculty of Medicine, Chulalongkorn University, Bangkok, 10330 Thailand; 4 Center of Excellence in Computational Molecular Biology, Faculty of Medicine, Chulalongkorn University, Bangkok, 10330 Thailand; 5 Division of Nutrition, Department of Pediatrics, King Chulalongkorn Memorial Hospital, The Thai Red Cross Society, Bangkok, 10330 Thailand; 6 Center of Excellence in Pediatric Nutrition, Division of Nutrition, Department of Pediatrics, Faculty of Medicine, Chulalongkorn University, 1873 Rama IV Road, Pathumwan, Bangkok, 10330 Thailand

**Keywords:** Gut-brain axis, Amino acids, Bioactive molecules, Biogenic amines, Inulin, Children with obesity, Biomarkers, Paediatric research, Medical research, Clinical trial design, Randomized controlled trials, Nutrition disorders, Obesity

## Abstract

The gut microbiota plays a key role in regulating energy balance via gut-brain axis (GBA). Dysbiosis can disrupt this communication, contributing to obesity. This study aimed to assess the effects of inulin supplementation on GBA-related amino acids and bioactive molecules in children with obesity. Children aged 7–15 were randomly assigned to 3 treatment groups for 6 months: inulin supplementation, isocaloric maltodextrin (placebo), or dietary fiber advice. Plasma amino acids and bioactive molecules were analyzed using LC-MS/MS at baseline and month 6. Relationships of changes in GBA-related compounds with changes in gut microbiota were evaluated. By month 6, principal component analysis trajectories showed clustering across all groups, involving 154 children, but indicated potential metabolic shifts, particularly in the inulin group. S-plots identified significant changes in GBA-related compounds, with only the inulin group showing marked increases in putrescine, spermine, and tyrosine from baseline (all *P* < 0.0001). Inulin supplementation significantly upregulated putrescine over time compared to the placebo group (*P* = 0.021), suggesting enhanced GBA communication. Changes in specific GBA-related compounds in the inulin group were significantly associated with gut microbiota changes. These findings indicate that inulin effectively modulates GBA-related bioactive molecules, potentially mediating its effect on childhood obesity management through putrescine, spermine, and tyrosine.

**Clinical Trial Registry number**: NCT03968003. Registered 30/05/2019.

**Supplementary Information:**

The online version contains supplementary material available at 10.1038/s41598-025-21079-2.

## Introduction

Obesity is a significant global health issue, with a strikingly high prevalence among children and adolescents^1^. These individuals are at increased risk for experiencing various comorbidities, including hypertension, obstructive sleep apnea, and metabolic syndrome^2,3^. Childhood and adolescent obesity frequently arises from an intricate combination of genetic, environmental, and lifestyle influences.

Emerging evidence suggests that gut microbiota can influence the host’s energy regulation, body composition, and appetite control^4–6^. Certain gut microbes and the microbial-derived compounds, such as tryptophan (Trp) metabolites, glutamate (Glu), γ-aminobutyric acid (GABA), dopamine, serotonin (5-HT), catecholamines, and histamine, may directly impact the central nervous system (CNS) via the gut-brain axis (GBA) by modifying key physiological mechanisms in those with obesity^7^. Several amino acids are precursors of neurotransmitter production. GABA, an inhibitory neurotransmitter in the CNS, produced by gut microbiota from the dietary amino acid Glu, plays a crucial role in gut-brain communication and subsequently in appetite regulation, as well as its connection to energy homeostasis and obesity^8,9^. Previous studies have also highlighted that 5-HT, produced by Trp, plays a role in modulating metabolism, while gut microbiota can influence both the production and release of 5-HT, affecting energy expenditure^10,11^. Dysregulation of 5-HT and its metabolites in adipose tissue is also linked to obesity and insulin resistance^12^. Dopamine is crucial for neurological functions and regulates gut motility and exocrine secretions. It is produced in both the brain and gastrointestinal tract, with tyrosine (Tyr) serving as its precursor. In obesity, altered dopamine signaling, including reduced D2 receptor binding in the striatum, is associated with reward-driven feeding behavior^13–15^. Additionally, changes in gut microbiota, specifically microbial dysbiosis, could affect dopaminergic transmission and gut-brain communication, potentially contributing to overeating^16^.

Given the above findings, the gut microbiota has emerged as a potential target for new obesity management strategies. Central to this is the microbiota-gut-brain axis (MGBA), where gut bacteria convert amino acids and biogenic amines into neuroactive metabolites that directly influence central appetite control by acting on hypothalamic pathways, thereby regulating appetite and energy balance. Dysregulation of the gut microbiota and their metabolites, however, can impair this crucial gut-brain communication, ultimately contributing to obesity^9,17^. It has been elucidated that inulin, a type of prebiotics, may offer a viable approach to address this dysregulation. Inulin could restore gut microbiota by promoting the growth of beneficial microbes, which in turn is hypothesized to increase the production of various GBA-related bioactive molecules. This leads to a more balanced and harmonious gut-microbiota interaction, thereby modulating the MGBA. This is thought to play a significant role in contributing to the management of childhood obesity. Therefore, this study aimed to assess the impacts of inulin supplementation on GBA-related amino acids and biogenic amines and evaluate the complex relationships of these GBA-related bioactive molecules with gut microbiota and clinical parameters in children with obesity.

## Materials and methods

### Study participants

This study was designed as a randomized, double-blind, placebo-controlled trial carried out from August 2017 to July 2020 at King Chulalongkorn Memorial Hospital (KCMH) in Thailand. A comprehensive description of the protocol can be found in a previous publication^18^. The study involving human participants, materials, and data adhered to the principles outlined in the Declaration of Helsinki. The protocol was approved by the Institutional Review Board of the Faculty of Medicine, Chulalongkorn University (IRB no. 240/60). Informed consent was obtained from all subjects and/or their legal guardian(s). The trial was registered on clinicaltrials.gov under the identifier NCT03968003 (Registered 30/05/2019). The inclusion and exclusion criteria were identical to those used in our previous study^18^. Briefly, children aged 7 to 15 years with obesity, defined as a body mass index (BMI) exceeding the median plus two standard deviations (SDs) based on the World Health Organization growth reference^1^, were eligible for inclusion. Exclusion criteria comprised syndromic or monogenic obesity, endocrine causes of obesity (e.g., hypothyroidism or growth hormone deficiency), use of medications known to affect appetite or body weight, allergy to inulin, and participation in other concurrent weight reduction programs.

### Study design

The complete study design is described elsewhere^18^. In summary, 165 participants were randomly divided into three groups. Participants in the inulin group consumed 13 g of extracted inulin powder from Thai Jerusalem artichoke daily for 6 months, approximately 30 min before dinner. The powder was mixed with 150 ml of warm water and stirred until dissolved. The placebo group consumed 11 g of isocaloric maltodextrin (Oligocarb; Ma-Jusmin Company Limited, Bangkok, Thailand) daily, using the same preparation method. The inulin extraction process was performed according to our patented method (Inventors: Chonnikant Visuthranukul and Supakarn Chamni, Chulalongkorn University and National Science and Technology Development Agency, Thailand, Thai Patent No. 15858, issued January 23, 2020). The third group was given dietary fiber guidance that included portion size illustrations to age-appropriate intake^19^. All participants were provided with the same general advice regarding diet, exercise, and behavior modification, along with monthly follow-ups for six months.

### Assessment of dietary intake, physical activity, anthropometry, and body composition

The full details are available in a previous publication^18^. In summary, a dietitian assessed dietary intake using 3-day dietary records, with daily energy and nutrient intake calculated via the Institute of Nutrition, Mahidol University Calculation-Nutrients (INMUCALs) Version 3^20^. Physical activity was evaluated through questionnaires. Trained personnel collected anthropometric and body composition data, following methods from the prior study^18^.

### AbsoluteIDQ^®^ p180 assay and sample Preparation

Blood was drawn at the KCMH after participants fasted overnight. Citrate vacutainers underwent centrifugation for 15 min at 1,500×g at room temperature. Following this, 120 µL aliquots were taken for plasma and preserved at -80 °C until use. The AbsoluteIDQ^®^ p180 kit, supplied by Biocrates Life Science AG (Innsbruck, Austria) and Helena Thai Laboratories co., Ltd (Bangkok, Thailand), included all essential reagents, internal and calibration standards, quality controls, test mix, and a 96-well filter plate. This kit employs an automated assay that utilizes phenylisothiocyanate (PITC) derivatization to analyze target analytes in biological fluids, with internal standards used for precise quantification. Amino acids and biogenic amines were detected using liquid chromatography-mass spectrometry (LC-MS). For plasma sample preparation, as per the manufacturer’s protocol, 10 µL of the sample was applied to the 96-well plate and dried under nitrogen. A 5% PITC solution (50 µL) was then added to derivatize the amino acids and biogenic amines, followed by another drying step. Metabolites were extracted using 300 µL of 5 mM ammonium acetate in methanol, transferred to the lower 96-well plate, and further diluted with MS solvent A (comprised 0.2% formic acid in water) for analysis. Quantification was based on 7 internal standards and a calibration curve with human plasma-based quality controls in 3 concentration levels (low, medium, high)^21^. The list of all amino acids and biogenic amines measured using the AbsoluteIDQ^®^ p180 Assay is provided in Supplementary Table S1. Our primary hypothesis for this study specifically focuses on the GBA, and therefore, our discussion and in-depth analyses have focused on compounds directly involved in this pathway, which were Glu, glutamine (Gln), histidine (His), histamine, Trp, Tyr, dopamine, putrescine, spermidine, spermine, and 5-HT.

### LC-MS

The LC-MS/MS configuration included an ACQUITY UPLC system (Waters, Milford, MA, USA) connected to a QTRAP5500 mass spectrometer (AB Sciex, Redwood City, CA, USA) operating in electrospray ionization (ESI) mode. This setup was used to analyze amino acids and biogenic amines in positive mode via LC-MS. A volume of 2 µL from the sample extract was applied to an ACQUITY BEH C18 column (2.1 × 7.5 mm, 1.7 μm), kept at a temperature of 50 °C. The method featured a 7.3-minute solvent gradient, where solvent A and solvent B contained 0.2% formic acid in acetonitrile.

### Inflammatory cytokines

The full details are described in a previous publication^22^. In brief, after a 12-hour fast, venous blood samples were collected to assess inflammatory cytokines, interleukin-1β (IL-1β), IL-6, and tumor necrosis factor-α (TNF-α) at baseline and month 6. Plasma inflammatory cytokines were measured with Bio-Plex Pro™ Human Cytokine Assays (Bio-Rad, USA) using the Bio-Plex Suspension Array System, based on Luminex xMAP technology.

### Fecal collection for 16S rRNA sequencing and short-chain fatty acid (SCFA) analysis

Baseline fresh fecal samples were collected for gut microbiota and SCFAs analysis, as described in a prior publication^5,23^. Participants received sterile collection kits and were instructed to gather samples at home. Each sample was placed in a 50 ml sterile container, sealed in a zip-lock bag, and stored in a home freezer (around -20 °C) before being delivered to the lab within 24 h. Upon receipt, samples were stored at -80 °C until analysis. Illumina paired-end reads of 16S rRNA were then analyzed as previously outlined. For SCFA analysis, fecal samples were 10-fold diluted with phosphate-buffered saline (pH 8.0) and homogenized using a stomacher blender (Stomacher^®^ 80 Biomaster; Seward, Worthing, UK) for 5 min, adapted from Kisuse et al.^24^. The resulting 1 mL fecal slurry was then centrifuged at 13,000×g for 5 min, with the supernatant stored at -80 °C until analysis. Lactic acid and SCFAs (acetic, butyric, and propionic acid) were quantified from these prepared samples via high-performance liquid chromatography (HPLC) (Water 1525, USA). HPLC sample preparation followed previously established, modified methodologies^25,26^, with all analytical parameters assessed using Agilent Technologies 7890 A equipment (Santa Clara, USA).

### Satiety hormone analysis

Participants provided 12-hour fasting serum samples, which were immediately placed on a test tube rack to clot at room temperature for 10-20 min. Within 30 min of collection, the blood underwent centrifugation at 1,820×g for 20 min. The resulting serum samples were then preserved at -80 °C until subsequent analysis. Levels of glucagon-like peptide 1 (GLP-1) and peptide YY (PYY) were determined using Human GLP-1 and Human Peptide YY Enzyme-Linked Immunosorbent Assay (ELISA) Kits (MyBioSource, Inc., San Diego, CA, USA), respectively. Both hormones were quantified using the Sandwich ELISA technique. An experienced technician performed the analysis and calculated results using computer-based curve-fitting software. The GLP-1 assay had a detection range of 15 to 3,000 ng/L with a sensitivity of 7.29 ng/L, while the PYY assay ranged from 3 to 900 pg/mL with a sensitivity of 1.56 pg/mL.

### Data analysis

A principal component analysis (PCA) was applied to amino acid and biogenic amine profiles at both baseline and after the intervention to visualize changes in the metabolite profiles across the placebo, inulin, and dietary fiber advice groups over the six-month period. To determine the statistical significance of the PCA, we applied the PCAtest function^27^, through random permutations to generate null distributions for key PCA statistics. These included testing whether the observed correlational structure among variables was random or not, determining the statistical significance of each principal component, and evaluating the contribution of each variable to the significant components. Bootstrap replicates were used to estimate sampling variance around the mean statistics. S-plots derived from the orthogonal partial least squares discriminant analysis (OPLS-DA) model identified the amino acid and biogenic amine profiles responsible for the largest separation between these metabolite profiles measured before (month 0) and after the intervention (month 6)^28^. Significant metabolites were identified in the three study groups over the six-month period using a VIP score greater than or equal to 1, *P* < 0.05, along with correlation less than -0.5 or greater than 0.5^28^.

The across-group differences between amino acid and biogenic amine profiles at baseline and the changes from month 0 to month 6 were assessed using Kruskal-Wallis (for baseline profiles) and one-way analysis of variance (ANOVA) (for month 0 to month 6 changes). To identify specific pairs of groups with significant differences in the changes of Putrescine from month 0 to month 6, a series of pairwise independent sample *t*-tests were performed.

All statistical analyses were performed using R statistical software^29^. PCA and OPLS-DA were conducted using *FactoMineR* and *ropls* packages, respectively. Data visualizations, including PCA trajectory plots and S-plots, were generated using *ggplot2*^30^.

Associations between GBA-related amino acid and biogenic amines, gut microbiota genus abundances, and clinical parameters were measured using Spearman’s rank correlation coefficient. P-values were first calculated based on the assumption that the parameters are normally distributed and uncorrelated, as implemented in the *spearmanr* function of the SciPy package^31^, and subsequently corrected for multiple testing with Benjamini-Hochberg procedure at 5% false discovery rate. Only significant associations were visualized in the circus plot, using *pycirclize* package^32^, and the network plot, using CytoScape^33^. It should be noted that not all measurements are normally distributed (tested using SciPy’s *normaltest* function with P-value cutoff of 0.5), but this assumption was used only to estimate the significance for Spearman’s rank correlation. The heatmaps of correlation coefficients (Supplementary Figs. S2 and  S4) are also provided for full transparency.

## Results

A total of 165 Thai children with obesity (mean age 10.4 ± 2.2 years, 59% male) were randomized into inulin, placebo, or dietary fiber advice groups. Of these, 154 participants who completed the study and provided plasma amino acid and biogenic amine data at baseline and the 6^th^ month were included in the analysis. Attrition rates were comparable between the groups, and no significant side effects were noted in the inulin or placebo groups^18^.

### Baseline demographic data, plasma amino acids, and biogenic amines

An overview of demographic data and baseline characteristics across all groups is provided in Supplementary Table S2, which has been reported previously^18^. No significant differences were observed in clinical data, nutrient intake, physical activity, or biochemical parameters. Additionally, baseline GBA-related plasma amino acids and biogenic amines showed no significant differences between the three groups (all *P* > 0.05) (Table 1).

**Table 1:** Baseline GBA-related plasma amino acids and biogenic amines in children with obesity.

Parameters	Placebo (*n* = 55)	Inulin (*n* = 55)	Dietary fiber advice (*n* = 55)
Amino acids			
Glutamate (µmol/L)	53.25 ± 15.43	52.08 ± 21.75	50.50 ± 21.38
Glutamine (µmol/L)	368.5 ± 153.6	387.5 ± 137.1	382.6 ± 123.4
Histidine (µmol/L)	52.61 ± 21.15	54.87 ± 18.02	53.94 ± 19.58
Tryptophan (µmol/L)	35.00 ± 15.20	36.88 ± 17.49	37.95 ± 13.92
Tyrosine (µmol/L)	45.85 ± 27.23	49.60 ± 31.46	48.85 ± 30.84
Biogenic amines			
Dopamine (µmol/L)	0.38 ± 0.22	0.37 ± 0.17	0.38 ± 0.24
Histamine (µmol/L)	0.37 ± 0.14	0.38 ± 0.14	0.38 ± 0.15
Putrescine (µmol/L)	0.083 ± 0.047	0.084 ± 0.048	0.086 ± 0.051
Serotonin (µmol/L)	0.072 ± 0.075	0.076 ± 0.087	0.093 ± 0.095
Spermidine (µmol/L)	0.25 ± 0.09	0.25 ± 0.06	0.26 ± 0.07
Spermine (µmol/L)	0.23 ± 0.09	0.22 ± 0.07	0.24 ± 0.07

Data shows mean ± SDs, All *P* > 0.05 by Kruskal-Wallis Test.

### PCA plots of amino acids and biogenic amines across the three groups over the 6-month intervention

**Fig. 1:**
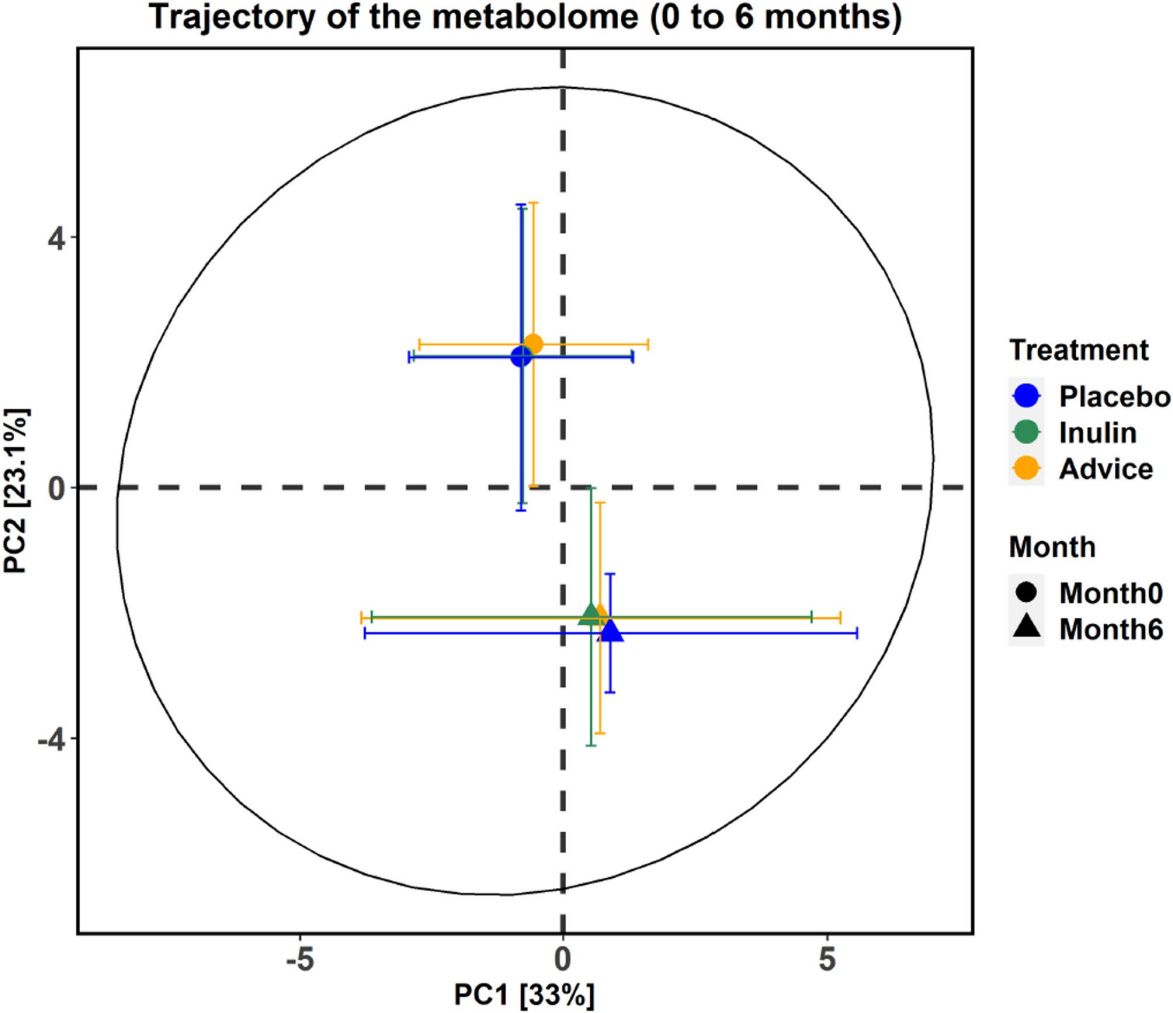
PCA plots of the amino acid and biogenic amine profiles. PCA trajectory plots of the amino acids and biogenic amines from baseline (month 0) to six months (month 6) across the placebo, inulin, and dietary fiber advice groups. The plots use color coding to differentiate the groups: blue represents the placebo group, green represents the inulin group, and orange represents the dietary fiber advice group. This shows group trajectories along principal components PC1 (33% explained variance) and PC2 (23.1% explained variance), indicating shifts in amino acids and biogenic amines over time. PCA, principal component analysis.

The PCA plots illustrate the changes in amino acids and biogenic amines between the placebo, inulin, and dietary fiber advice groups over the 6-month intervention (Fig. 1). A permutation-based test was used to determine the significance of PCA model, using 1000 permutations and 1,000 bootstrap iterations through the PCAtest function. The test indicated that the first three principal components were statistically significant, accounting for 61.3% of the total variation. PC1 accounted for 33.0% of the total variation (95% CI: 31.9-34.8), PC2 explained 23.1% (95% CI: 21.3-26.2), and PC3 contributed 5.3% (95% CI: 4.8-6.2). As per the permutation test, 33 metabolites were found to significantly load PC1 and PC2, while five were found to significantly load PC3 (Supplementary Table S3). For a given month, the three study groups were clustered together, with profiles measured at month 6 exhibiting a pronounced shift from that measured at month 0. Additionally, the deviation along PC2 (indicated by error bars) at month 6 was also larger than that at month 0. The loading plots for individual amino acids and biogenic amines for the first two principal axes (PC1 and PC2) are shown in Supplementary Fig. S1.

### OPLS-DA of amino acids and biogenic amines over the 6-month intervention

**Fig. 2:**
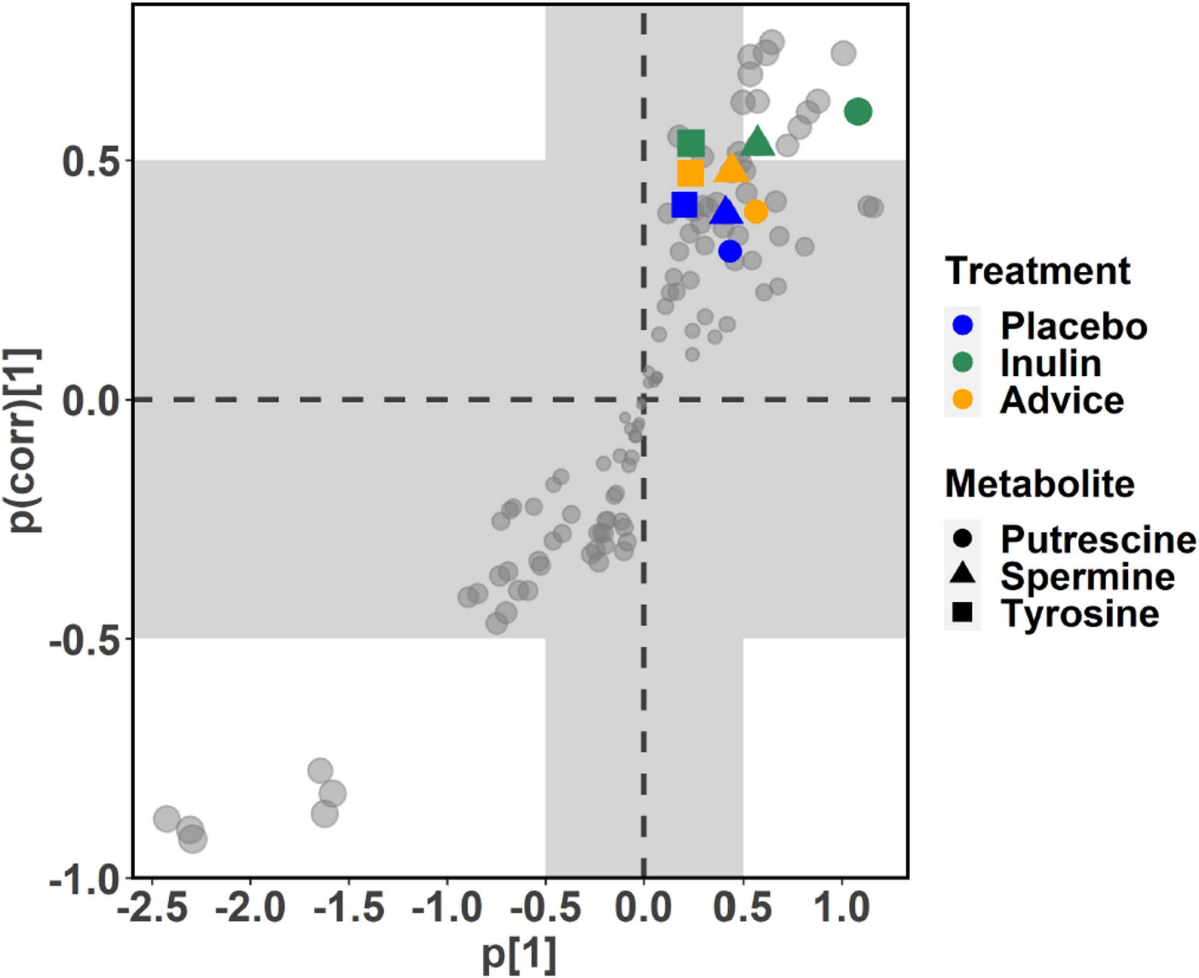
Changes in amino acids and biogenic amines within each study group over the six-month intervention, as determined by the S-plots from OPLS-DA analysis. S-plots were a visual representation of metabolite loadings, aiding in the identification of compounds highly correlated with interventions. Among the significant compounds, putrescine (filled circles), spermine (filled triangles), and Tyr (filled squares) are highlighted according to their respective groups, with blue for placebo, green for inulin, and orange for dietary fiber advice. The inulin group demonstrated a significant increase in putrescine, spermine, and Tyr from baseline to month 6 (all *P* < 0.0001), whereas no significant changes were observed in the other two groups. OPLS-DA, orthogonal partial least squares discriminant analysis; Tyr, tyrosine.

S-plots derived from OPLS-DA were used to identify key amino acids and biogenic amines within each study group over the 6-month period. Significant changes were observed, particularly an enhancement of GBA-related amino acid and biogenic amines in the inulin group. Specifically, Tyr showed a significant change (VIP score = 1.244, correlation P-value: 9.58E-09). Similarly, putrescine (VIP score = 1.396, correlation P-value: 3.98E-11) and spermine (VIP score = 1.236, correlation P-value: 1.25E-08) also demonstrated significant changes within the inulin group during this period. No significant changes were noted in the placebo and dietary fiber advice groups (Fig. 2). The complete results for all compounds from these within-group analyses are presented in Supplementary Table S4.

**Fig. 3:**
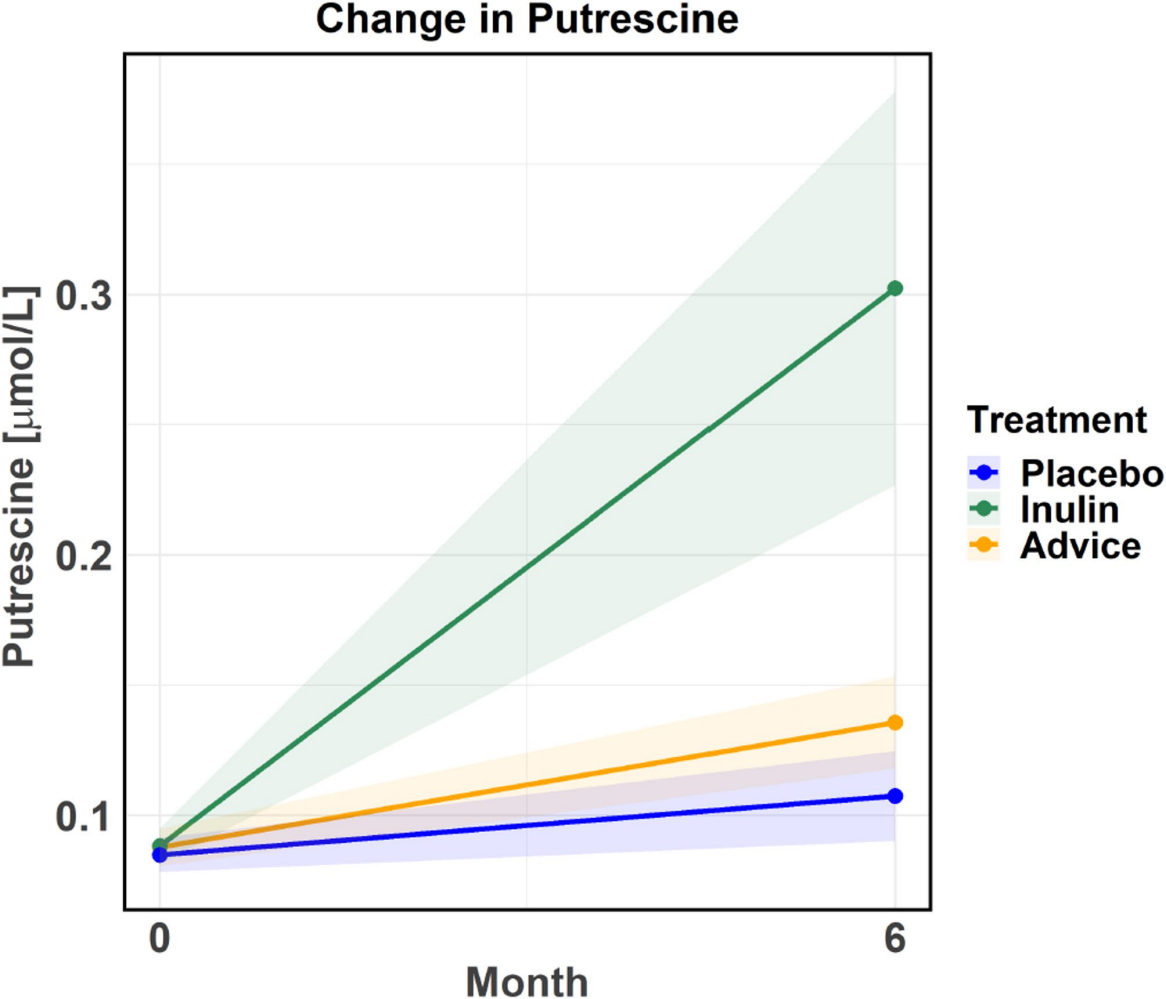
Change in putrescine across the placebo, inulin, and dietary fiber advice groups over the 6-month intervention. The plot uses color coding to differentiate the groups: blue represents the placebo group, green represents the inulin group, and orange represents the dietary fiber advice group. A one-way ANOVA test was performed (*P* < 0.01), followed by post-hoc pairwise independent sample t-tests, which revealed that inulin supplementation significantly upregulated putrescine over time compared to the placebo group (*P* = 0.021). Bonferroni correction was applied account for multiple comparisons (time and treatment). ANOVA, analysis of variance.

Notably, inulin supplementation significantly upregulated putrescine levels over time compared to the placebo group (one-way ANOVA *P* < 0.01, *t*-test *P* = 0.021) (Fig. 3). Putrescine levels over the 6-month period across the three groups are presented as mean (MeanZScore), standard deviation (SD), and standard error (SE). The mean values are plotted over time with ± SE ribbons, where SE was calculated as SE = SD/(√N), indicating the deviation around the mean estimates. A pairwise t-test was used to compare putrescine levels between groups. The Bonferroni correction was applied to the P-values to correct for multiple comparisons (time and intervention). All compounds from the between-group comparisons are presented in Supplementary Table S5.

### Relationships of GBA-related amino acids and biogenic amines with gut microbiota at baseline

**Fig. 4:**
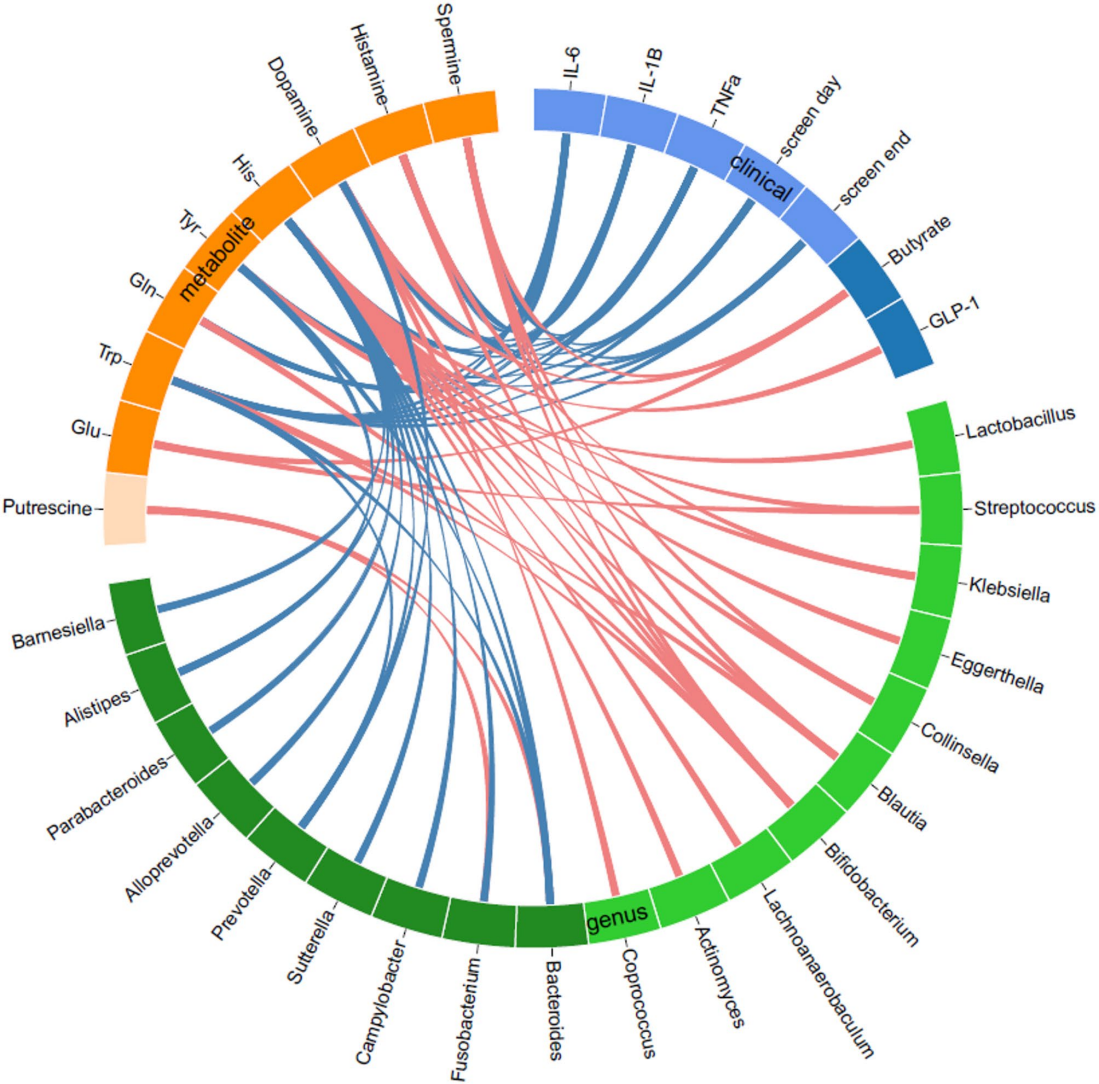
Relationships of GBA-related amino acids and biogenic amines with gut microbiota, physical activity, and biochemical parameters at baseline by circos plot. Red lines indicate positive associations, and blue lines indicate negative associations, analyzed by Spearman’s correlation coefficient with Benjamini-Hochberg correction with FDR = 0.05. GBA, gut-brain axis; Gln, glutamine; Glu, glutamate; GLP-1, glucagon-like peptide 1; His, histidine; IL-1β, interleukin-1β; IL-6, interleukin-6; Screen day, screen time on weekdays; Screen end, screen time on weekends; TNFa, tumor necrosis factor-α; Trp, tryptophan; Tyr, tyrosine.

Among amino acids, Glu positively correlated with *Streptococcus*, Gln with *Bifidobacterium*, and His with *Bifidobacterium*,* Collinsella*,* Blautia* and *Streptococcus*, but negatively with *Parabacteroides*, *Fusobacterium*, and *Prevotella*. Trp was linked to *Bifidobacterium* and *Blautia*, but inversely to *Prevotella*. Tyr was positively associated with *Lactobacillus* but negatively associated with *Alistipes.* For biogenic amines, dopamine positively correlated with *Bifidobacterium* but negatively with *Prevotella*. Putrescine showed positively related to *Bacteroides* and *Fusobacterium*, and spermine to *Bifidobacterium* and *Blautia* (all *P* < 0.05 after Benjamini-Hochberg correction with FDR = 0.05) (Fig. 4 and Supplementary Fig. S2).

### Relationships of GBA-related amino acids and biogenic amines with clinical parameters at baseline

Glu positively correlated with butyrate, while His had a positive relationship with GLP-1. Trp and Tyr negatively related to screen time. His, Gln, and Trp demonstrated negative correlations with the inflammatory cytokines, IL-1β, IL-6, and TNF-α. For biogenic amines, dopamine also negatively correlated with screen time and these cytokines but had a positive association with butyrate. Spermine was positively related to butyrate but negatively related to screen time and TNF-α. (all *P* < 0.05 after Benjamini-Hochberg correction with FDR = 0.05) (Fig. 4 and Supplementary Fig. S3).

### Relationships of changes in GBA-related amino acids and biogenic amines with changes in gut microbiota after intervention

Following intervention, we observed different associations in the changes in metabolites and gut microbiota both across groups (Supplementary Fig. S4) and with the baseline (Supplementary Fig. S2). Within the inulin supplementation group, there were positive correlations between changes in putrescine with changes in gut microbiota such as *Bacteroides* and *Olsenella*. Changes in Trp, His, and spermine were positively correlated with changes in *Chloroplast*, and *Peptostreptococcus*, but negatively correlated with changes in *Prevotella* and *Oscillibacter* for example. However, some of these associations are either lost or reverted in the placebo or dietary advice groups.

**Fig. 5:**
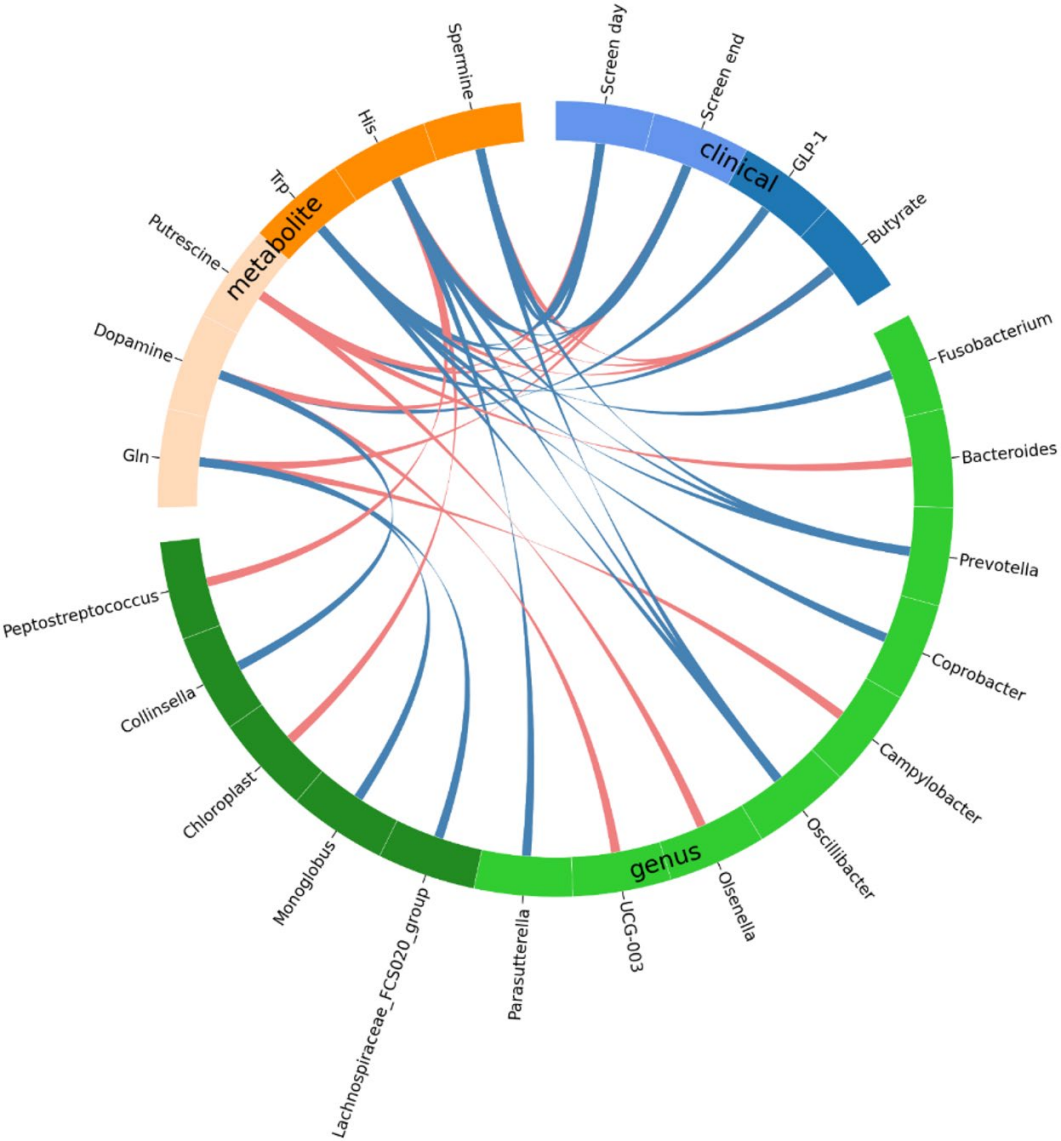
Relationships of the changes (month 0 to month 6) in GBA-related amino acids and biogenic amines with the changes (month 0 to month 6) in gut microbiota, physical activity, and biochemical parameters by circos plot. Red lines indicate positive associations, and blue lines indicate negative associations, analyzed by Spearman’s correlation coefficient with Benjamini-Hochberg correction with FDR = 0.05. GBA, gut-brain axis; Gln, glutamine; GLP-1, glucagon-like peptide 1; His, histidine; Screen day, screen time on weekdays; Screen end, screen time on weekends; Trp, tryptophan.

There were consistent associations between putrescine and gut microbiota that were observed both at baseline and after treatment. For example, as high baseline putrescine was associated with high baseline *Bacteroides* and *Fusobacterium* (Fig. 4), increase in putrescine was also associated with increase in *Bacteroides* following inulin supplementation (Fig. 5 and Supplementary Fig. S4).

## Discussion

To our knowledge, this is the largest RCT to examine the effects of prebiotic on GBA-related amino acids and biogenic amines in children with obesity. Inulin supplementation significantly upregulated putrescine over time compared to the other groups. Putrescine, spermine, and Tyr, also showed significant changes from baseline to the 6th month, exclusively in the inulin group. Specific GBA-related bioactive molecules demonstrated distinct associations with gut microbiota, clinical parameters, and metabolic biomarkers in children with obesity.

Polyamines are produced through the decarboxylation of amino acids and play a crucial role in supporting cell growth and development by initiating various cellular responses^34^. They are an integral component of cellular and genetic metabolism, and the concentration of polyamines within cells can also influence the progression of various diseases^35–37^. Putrescine, a polyamine produced by gut bacteria, emerged as a crucial biomarker in our study, especially in the context of prebiotic intervention. A substantial elevation in putrescine was found after inulin supplementation compared to the other groups, highlighting its potential role in mediating the beneficial effects of inulin on obesity outcomes. As the most abundant polyamine in the intestine, putrescine is rapidly absorbed and converted into spermidine and spermine, which exert stronger physiological effects^38^. Notably, putrescine activates histamine receptors^39^, contributing to the regulation of appetite and satiety, which are key mechanisms involved in obesity management. Therefore, the effect of inulin on obesity outcomes could be mediated through putrescine. Beyond its role in energy balance, putrescine also supports vital cellular activities, including signaling, DNA interactions, transcription processes, and RNA splicing, similar to spermine^40,41^. Moreover, spermine, a biogenic amine, serves as a precursor for enzymes such as spermine oxidase and polyamine oxidases, which are integral to polyamine metabolism and have significant implications for health^42,43^. Importantly, spermine also modulates glutamate receptors and influences neurotransmitter release, contributing to the optimization of appetite-regulating circuits and linking microbial metabolism to central appetite control. In our study, significant modulation of both putrescine and spermine was found exclusively in the inulin group over time, indicating their potential role in gut-brain signaling and metabolic regulation through neurotransmitter pathways. In addition, inulin supplementation significantly increased Tyr, a dopamine precursor that plays a critical role in controlling appetite and maintaining energy balance. Disruptions in gut-brain communication in obesity, which are characterized by altered hormone signaling, inflammation, and gut dysbiosis, are associated with impaired Tyr-dopamine pathways^16,44–46^, contributing to dysregulated eating behaviors. The rise in Tyr levels following inulin intervention points to its potential role in restoring dopaminergic signaling in children with obesity. Taken together, these findings suggest that the beneficial effects of inulin on obesity outcomes might be mediated through the modulation of spermine and Tyr. Both metabolites have established roles in appetite regulation and neural communication. These results provide novel insights into the mechanisms by which inulin may influence host metabolism and enhance satiety signaling. Importantly, this is the first randomized controlled trial in children with obesity to report such findings, underscoring the innovative contribution of this study. As we observed, specific GBA-related bioactive molecules exhibited unique associations with gut microbiota, clinical and metabolic parameters in children with obesity. Glu is a key amino acid, and certain intestinal bacteria convert Glu into GABA via glutamate decarboxylase^47^. Genera like *Lactobacillus*, *Bifidobacterium*, and *Streptococcus* are known to produce GABA, the main inhibitory neurotransmitter in the CNS, which plays a role in regulating metabolism and obesity^8^. Some *Bifidobacterium* strains may biosynthesize GABA through Gln, which impacts central energy metabolism^48^. Gln is then converted back to Glu in neurons, essential for neurotransmission and neuronal development. Consistent with these processes, our study found that Glu was positively related to *Streptococcus*, while Gln was linked to *Bifidobacterium*, highlighting their role in gut-brain communication. The study by Quesada-Vázquez et al. found that circulating His was positively associated with gut bacteria, such as *Faecalibacterium* and *Bifidobacterium*, similar to our findings^49^. Histamine, synthesized from His, serves important physiological functions, including feeding regulation^50^. Gut microbiota like *Lactobacillus* spp. produces histamine, consistent with our result, to communicate with the CNS^51^. Recent study emphasized the essential role of Trp in facilitating communication between gut microbiota and the brain^52^. Certain bacteria, particularly *Bifidobacterium* and *Blautia*, demonstrated beneficial impacts on Trp processing. In one study, administering probiotic *Bifidobacterium infantis* to rats led to increased circulating Trp, which could enhance 5-HT synthesis in the brain, potentially regulating appetite control and digestion^53^. Tyr was negatively correlated with *Bacteroides* and *Alistipes*, consistent with a previous study^54^. Tyr serves as the building block for the neurotransmitter dopamine.

Dopamine was positively correlated with *Bifidobacterium*, but negatively with *Prevotella* in our findings. Previous studies show that gut microbes like *Bifidobacterium*, *Bacteroides*, *Lactobacillus*, and *Prevotella* could modulate dopaminergic pathways by affecting receptors and transporters^12,55^. Disruptions in dopamine can impair hypothalamic pathways controlling satiety, contributing to excessive weight gain and obesity development^56^. Moreover, in terms of biogenic amines, *Bacteroides* spp. and *Fusobacterium* spp. have been shown to produce putrescine both in vitro^57^ and in vivo^58^. Our study further revealed a positive correlation between putrescine levels and these genera. Notably, this relationship was observed not only at baseline but also in response to inulin supplementation. Specifically, higher baseline levels of putrescine were associated with higher abundances of *Bacteroides* and *Fusobacterium*, and an increase in putrescine levels after treatment was paralleled by an increase in *Bacteroides*. These findings reinforce the potential role of *Bacteroides*, in particular, in putrescine production over time. This aligns with evidence that the concentrations of biogenic amines putrescine and spermidine in the intestinal lumen are largely regulated by the colonic microbiota, which may also interact with neuronal receptors and influence gut-brain signaling^59^. Further illustrating the complex microbiota-metabolite interactions, we found that Trp levels were negatively correlated with *Prevotella* abundance at both baseline and after inulin supplementation. Notably, participants with lower baseline Trp levels exhibited higher *Prevotella* abundance, while increases in Trp following the intervention were associated with a decrease in *Prevotella*, an effect observed only in the inulin group. This pattern may reflect a beneficial metabolic shift relevant to childhood obesity management. These findings are consistent with a recent study reporting that a higher *Prevotella*/*Bacteroides* (P/B) ratio was associated with lower fecal Trp concentrations and increased centrality of the nucleus accumbens, a key region in the brain’s reward network^60^. Greater centrality in this region has been linked to heightened reward sensitivity, potentially leading to food cravings and overeating. Thus, lower Trp levels associated with a higher P/B ratio could influence serotonergic pathways involved in appetite regulation, contributing to dysregulated eating and obesity. Our findings suggest that inulin supplementation may modulate gut microbiota-Trp interactions in a manner that influences these neural pathways. The fact that this association was evident only in the inulin group supports the idea that inulin enhances the functional expression of existing microbiota-metabolite interactions, rather than altering microbial abundance alone. Overall, inulin may regulate specific microbial pathways involved in amino acid and biogenic amine metabolism, with broader implications for microbiota-host communication and metabolic health in children with obesity via the GBA.

Interestingly, we found specific relationships of GBA-related amino acids and biogenic amines with some clinical and metabolic parameters. In our findings, Glu was positively correlated with butyrate. Glu produces butyrate, which enters the bloodstream to support neuroprotection and the development and function of brain microglia^61^. His may modulate GLP-1 secretion^62^, as shown by their positive correlation in our findings. Moreover, histamine plays a role in immunoregulation by inhibiting IL-18 production in the gut^63^, which demonstrated a negative association of His and histamine with inflammatory cytokines. We found that Trp and Tyr were negatively associated with a sedentary lifestyle. In previous study, physical exercise seems to promote intestinal bacteria-driven biosynthesis of Trp and Tyr^64^, which, in turn, influence gastrointestinal functions.

Our findings also suggest that physical activity may enhance dopaminergic function, as indicated by a negative correlation between dopamine and sedentary behavior. Additionally, dopamine released in the gut by microbial activity can regulate cytokine production. Our study found a negative correlation between dopamine and inflammatory cytokines, suggesting a role of dopamine in modulating the inflammatory response. A positive correlation between spermine and butyrate and a negative association with sedentary lifestyle were observed and spermidine was negatively related to TNF-α. These findings support the diverse roles of biogenic amines in intestinal barrier maintenance and anti-inflammatory effects^65^.

### Strengths and limitations

The present study is the largest and first randomized, double-blinded, placebo-controlled trial to investigate the effects of prebiotic supplementation on GBA-related amino acids and bioactive molecules in children with obesity. By demonstrating that inulin significantly enhanced key markers of gut-brain communication, including putrescine, spermine, and Tyr. These findings highlight the potential of inulin as a targeted intervention for managing pediatric obesity by improving gut-brain signaling. Furthermore, the use of advanced technique, specifically LC-MS/MS, ensured the depth and robustness of the analyses. The limitations of the study include its 6-month duration, which may not capture the long-term effects of inulin supplementation on gut-brain communication and clinical outcomes. Additionally, while the study identified changes in GBA-related compounds, detailed mechanistic pathways were not directly explored. Addressing these aspects in future research could provide a more comprehensive understanding.

## Conclusions

In conclusion, inulin supplementation significantly enhanced putrescine, spermine, and Tyr over time, suggesting its potential influence on gut-brain communication and metabolic regulation in obesity. Moreover, several GBA-related amino acids and biogenic amines were significantly associated with gut microbiota composition, lifestyle activity, and biochemical parameters. These findings highlight the complexity of MGBA interactions in regulating amino acids, biogenic amines, and neurotransmitters. Future studies should explore the specific mechanisms through which these bioactive molecules contribute to MGBA regulation and their potential as therapeutic targets for managing childhood obesity.

## Supplementary Information

Below is the link to the electronic supplementary material.

Supplementary Material 1

Supplementary Material 2

## Data Availability

Data described in the manuscript will be made available upon request pending application and approval from the corresponding author.
