# Supplementary material for: Inulin supplementation modulates gut microbiota derived metabolites related to brain function in children with obesity
Source: Sci Rep. 2025 Oct 7;15:34843. doi: 10.1038/s41598-025-21079-2 (PMC12504693; doi:10.1038/s41598-025-21079-2)
Supplement: Supplementary file 1 — Supplementary Material 1 [file 41598_2025_21079_MOESM1_ESM.docx]

**Supplementary Table S1** List of all amino acids and biogenic amines measured using the AbsoluteIDQ^®^ p180 Assay. Complete list of amino acids and biogenic amines quantified in the study using the AbsoluteIDQ^®^ p180 targeted metabolomics platform.

| **Abbreviation** | **Compound class** | **Analyte** |
| --- | --- | --- |
| Ala | Amino acids | Alanine |
| Arg |  | Arginine |
| Asp |  | Aspartate |
| Cit |  | Citrulline |
| Gln |  | Glutamine |
| Glu |  | Glutamate |
| Gly |  | Glycine |
| His |  | Histidine |
| Ile |  | Isoleucine |
| Leu |  | Leucine |
| Lys |  | Lysine |
| Met |  | Methionine |
| Orn |  | Ornithine |
| Phe |  | Phenylalanine |
| Pro |  | Proline |
| Thr |  | Threonine |
| Trp |  | Tryptophan |
| Tyr |  | Tyrosine |
| Val |  | Valine |
| Ac-Orn | Biogenic amines | Acetylornithine |
| ADMA |  | Asymmetric dimethylarginine |
| SDMA |  | Symmetric dimethylarginine |
| alpha-AAA |  | alpha-Aminoadipic acid |
| Histamine |  | Histamine |
| Met-SO |  | Methionine-Sulfoxide |
| Putrescine |  | Putrescine |
| Spermidine |  | Spermidine |
| Spermine |  | Spermine |
| Serotonin |  | Serotonin |
| PEA |  | Phenylethylamine |
| Nitro-Tyr |  | Nitrotyrosine |
| c4-OH-Pro |  | cis-4-Hydroxyproline |
| t4-OH-Pro |  | trans-4-Hydroxyproline |
| Creatinine |  | Creatinine |
| Carnosine |  | Carnosine |
| Taurine |  | Taurine |
| DOPA |  | Dihydroxyphenylalanine |
| Dopamine |  | Dopamine |

**Supplementary Table S2** Baseline characteristics of children with obesity at the first visit (n = 165)

|  | **Placebo group**  **(n = 55)** | **Inulin group**  **(n = 55)** | **Dietary fiber advice group (n = 55)** |
| --- | --- | --- | --- |
| Age, years | 10.7 ± 2.4 | 10.3 ± 2.1 | 10.4 ± 2.0 |
| Male gender, % | 56.4 | 54.6 | 67.3 |
| Total nutrient intake |  |  |  |
| Energy intake, kcal/day | 1,419 ± 537 | 1,470 ± 571 | 1,463 ± 513 |
| Protein intake, g/kg/day | 1.53 ± 0.70 | 1.54 ± 0.57 | 1.67 ± 0.66 |
| Dietary fiber, g/1,000 kcal | 2.8 ± 1.9 | 2.9 ± 2.3 | 2.6 ± 1.9 |
| Fat intake, g/day | 56.9 ± 28.0 | 60.9 ± 31.1 | 58.3 ± 27.4 |
| Cholesterol intake, mg/day | 300 ± 198 | 316 ± 250 | 330 ± 236 |
| Energy distribution, %C: P: F | 48: 16: 36 | 48: 16: 36 | 47: 17: 36 |
| Exercise | | | |
| Low intensity, min/wk^1^ | 100 (10, 150) | 60 (0, 180) | 70 (0, 180) |
| Moderate intensity, min/wk^2^ | 75 (20, 150) | 60 (30, 150) | 30 (0, 75) |
| Sedentary activity, hr/day | 4.4 ± 3.0 | 4.5 ± 3.0 | 4.6 ± 3.1 |
| BMI, kg/m^2^ | 28.5 ± 4.6 | 28.3 ± 4.5 | 27.4 ± 3.4 |
| BMI for age z-score | 3.2 ± 1.1 | 3.3 ± 1.0 | 3.2 ± 0.95 |
| Waist circumference, cm | 90.6 ± 10.8 | 89.9 ± 11.3 | 88.6 ± 10.0 |
| SBP, mmHg | 115.3 ± 10.0 | 114.1 ± 8.3 | 117.4 ± 11.1 |
| Acanthosis nigricans, % | 83.6 | 76.4 | 80.0 |
| Tanner stage | | | |
| Stage 1, % | 56.36 | 65.45 | 69.09 |
| Stage 2, % | 10.91 | 20 | 12.73 |
| Stage 3, % | 20 | 12.73 | 10.91 |
| Stage 4, % | 10.91 | 1.82 | 7.27 |
| Stage 5, % | 1.82 | 0 | 0 |
| Body composition (measured by BIA) | | | |
| FMI, kg/m^2^ | 12.0 ± 2.9 | 11.9 ± 3.1 | 11.4 ± 2.7 |
| FFMI, kg/m^2^ | 16.31 ± 2.61 | 16.18 ± 1.90 | 15.98 ± 1.72 |
| Trunk FMI, kg/m^2^ | 5.7 ± 1.4 | 5.7 ± 1.5 | 5.5 ± 1.3 |
| VFA, cm^2^ | 133.9 ± 39.3 | 128.8 ± 42.0 | 125.3 ± 39.7 |

Data shows means ± SD, median (Q1, Q3) or %. One-way ANOVA was used to evaluate parametric variables, Kruskal-Wallis test was used to evaluate non-parametric variables, and Chi-square was used to evaluate categorical variables. No statistically significant between group difference was demonstrated for all parameters.

^1^Low intensity (min/wk) was walking from home to school or walking from one place to another for at least 10 minutes. ^2^Moderate intensity (min/wk) was brisk walking or riding a bicycle continuously for at least 10 minutes. Sedentary activity was defined as a type of lifestyle involving little or no physical activity.

BIA, bioelectrical impedance analysis; BMI, body mass index; FMI, fat mass index = fat mass (kg)/height (m^2^); FFMI, fat-free mass index = fat-free mass (kg)/height (m^2^); SBP, systolic blood pressure; VFA, visceral fat area.

**Supplementary Table S3** Results of principal component analysis (PCA) significance testing using permutation-based methods. Statistical significance of the principal components and contributions of individual amino acids and biogenic amines, based on the PCAtest function with 1,000 permutations and 1,000 bootstrap iterations.

| **PC1** | **PC2** | **PC3** |
| --- | --- | --- |
| Alanine | Alanine | Glycine |
| Arginine | Arginine | alpha-AAA |
| Aspartate | Aspartate | Carnosine |
| Citrulline | Glutamate | Serotonin |
| Glutamine | Glycine | Valine |
| Glutamate | Histidine |  |
| Glycine | Isoleucine |  |
| Histidine | Leucine |  |
| Leucine | Lysine |  |
| Lysine | Methionine |  |
| Methionine | Ornitine |  |
| Ornitine | Phenylalanine |  |
| Phenylalanine | Taurine |  |
| Proline | Threonine |  |
| Taurine | Tryptophan |  |
| Threonine | Ac-Orn |  |
| Tryptophan | ADMA |  |
| Tyrosine | alpha-AAA |  |
| Ac-Orn | c4-OH-Pro |  |
| ADMA | Carnosine |  |
| alpha-AAA | Creatinine |  |
| Carnosine | DOPA |  |
| Creatinine | Dopamine |  |
| DOPA | Histamine |  |
| Dopamine | Met-SO |  |
| PEA | Nitro-Tyr |  |
| Putrescine | PEA |  |
| SDMA | Putrescine |  |
| Serotonin | SDMA |  |
| Spermidine | Serotonin |  |
| Spermine | Spermidine |  |
| t4-OH-Pro | Spermine |  |
| Valine | Valine |  |

Ac-Orn, acetylornithine; ADMA, asymmetric dimethylarginine; alpha-AAA, alpha-aminoadipic acid; c4-OH-Pro, cis-4-hydroxyproline; DOPA, dihydroxyphenylalanine; Met-SO, methionine-sulfoxide; Nitro-Tyr, nitrotyrosine; PEA, phenylethylamine; SDMA, symmetric dimethylarginine; t4-OH-Pro, trans-4-hydroxyproline.

**Supplementary Table S4** Within-group analysis of amino acid and biogenic amine changes over the 6-month period. VIP scores and P-values for amino acids and biogenic amines showing significant changes within each group over time, based on orthogonal partial least squares discriminant analysis (OPLS-DA) and correlation analyses.

| **Metabolite** | **Correlation** | **P_corr** | **VIP** | **Treatment** | **Sig.corr** | **Sig.P.VIP** |
| --- | --- | --- | --- | --- | --- | --- |
| Ac-Orn | 0.56872847 | 3.01E-10 | 1.48320254 | Placebo | Significant | Significant |
| Ac-Orn | 0.62276831 | 1.68E-12 | 1.54399633 | Advice | Significant | Significant |
| Ac-Orn | 0.72300631 | 2.00E-17 | 1.68068758 | Inulin | Significant | Significant |
| ADMA | 0.36752384 | 0.00012407 | 0.9584755 | Placebo | Not Significant | Not Significant |
| ADMA | 0.41122323 | 1.45E-05 | 1.01952388 | Advice | Not Significant | Significant |
| ADMA | 0.40170499 | 3.44E-05 | 0.93379625 | Inulin | Not Significant | Not Significant |
| Alanine | -0.2671374 | 0.00611858 | 0.6966749 | Placebo | Not Significant | Not Significant |
| Alanine | -0.3164979 | 0.0010633 | 0.7846765 | Advice | Not Significant | Not Significant |
| Alanine | -0.297428 | 0.00265399 | 0.6913959 | Inulin | Not Significant | Not Significant |
| alpha-AAA | -0.2540832 | 0.00924945 | 0.66263059 | Placebo | Not Significant | Not Significant |
| alpha-AAA | -0.2315122 | 0.01804677 | 0.573976 | Advice | Not Significant | Not Significant |
| alpha-AAA | -0.2258291 | 0.02387296 | 0.52495831 | Inulin | Not Significant | Not Significant |
| Arginine | 0.2244289 | 0.02199974 | 0.58529428 | Placebo | Not Significant | Not Significant |
| Arginine | 0.3928373 | 3.71E-05 | 0.97394062 | Advice | Not Significant | Not Significant |
| Arginine | 0.50695454 | 7.36E-08 | 1.1784575 | Inulin | Significant | Significant |
| Aspartate | -0.3401526 | 0.00041099 | 0.88709329 | Placebo | Not Significant | Not Significant |
| Aspartate | -0.2780351 | 0.00426701 | 0.68931767 | Advice | Not Significant | Not Significant |
| Aspartate | -0.360142 | 0.00023283 | 0.8371796 | Inulin | Not Significant | Not Significant |
| c4-OH-Pro | -0.2242821 | 0.02208896 | 0.58491141 | Placebo | Not Significant | Not Significant |
| c4-OH-Pro | -0.1613891 | 0.10169139 | 0.40012341 | Advice | Not Significant | Not Significant |
| c4-OH-Pro | -0.1779514 | 0.07650949 | 0.41366277 | Inulin | Not Significant | Not Significant |
| Carnosine | -0.8235808 | 7.24E-27 | 2.14783886 | Placebo | Significant | Significant |
| Carnosine | -0.8661088 | 1.74E-32 | 2.14729751 | Advice | Significant | Significant |
| Carnosine | -0.7758849 | 2.55E-21 | 1.80360832 | Inulin | Significant | Significant |
| Citrulline | 0.34255656 | 0.00037155 | 0.89336264 | Placebo | Not Significant | Not Significant |
| Citrulline | 0.40535972 | 1.97E-05 | 1.00498679 | Advice | Not Significant | Significant |
| Citrulline | 0.34680343 | 0.00040808 | 0.80617308 | Inulin | Not Significant | Not Significant |
| Creatinine | -0.0760653 | 0.44281136 | 0.19837284 | Placebo | Not Significant | Not Significant |
| Creatinine | 0.03452116 | 0.72791757 | 0.08558648 | Advice | Not Significant | Not Significant |
| Creatinine | -0.056178 | 0.57878817 | 0.13059037 | Inulin | Not Significant | Not Significant |
| DOPA | 0.22409186 | 0.02220503 | 0.58441529 | Placebo | Not Significant | Not Significant |
| DOPA | 0.15644245 | 0.1127608 | 0.38785945 | Advice | Not Significant | Not Significant |
| DOPA | 0.23576189 | 0.01820767 | 0.54804788 | Inulin | Not Significant | Not Significant |
| Dopamine | 0.41332985 | 1.30E-05 | 1.07793423 | Placebo | Not Significant | Significant |
| Dopamine | 0.34083961 | 0.00039934 | 0.84502551 | Advice | Not Significant | Not Significant |
| Dopamine | 0.2884835 | 0.00360601 | 0.6706036 | Inulin | Not Significant | Not Significant |
| Glutamate | -0.120646 | 0.22248625 | 0.31463591 | Placebo | Not Significant | Not Significant |
| Glutamate | -0.0055047 | 0.95577256 | 0.01364757 | Advice | Not Significant | Not Significant |
| Glutamate | 0.05767178 | 0.56871723 | 0.13406279 | Inulin | Not Significant | Not Significant |
| Glutamine | 0.17210583 | 0.08063915 | 0.44883949 | Placebo | Not Significant | Not Significant |
| Glutamine | 0.3881216 | 4.68E-05 | 0.96224922 | Advice | Not Significant | Not Significant |
| Glutamine | 0.54946185 | 3.22E-09 | 1.27726923 | Inulin | Significant | Significant |
| Glycine | 0.13602879 | 0.16855252 | 0.35475322 | Placebo | Not Significant | Not Significant |
| Glycine | 0.19475185 | 0.04758062 | 0.4828379 | Advice | Not Significant | Not Significant |
| Glycine | 0.25642165 | 0.01001859 | 0.59607319 | Inulin | Not Significant | Not Significant |
| Histamine | -0.9190409 | 4.91E-43 | 2.39679183 | Placebo | Significant | Significant |
| Histamine | -0.8998906 | 1.52E-38 | 2.23105085 | Advice | Significant | Significant |
| Histamine | -0.8763262 | 7.50E-33 | 2.03709234 | Inulin | Significant | Significant |
| Histidine | 0.04655618 | 0.63885661 | 0.12141514 | Placebo | Not Significant | Not Significant |
| Histidine | -0.1374681 | 0.16405026 | 0.34081744 | Advice | Not Significant | Not Significant |
| Histidine | -0.0493237 | 0.62602269 | 0.11465699 | Inulin | Not Significant | Not Significant |
| Isoleucine | -0.3140662 | 0.00116747 | 0.81906196 | Placebo | Not Significant | Not Significant |
| Isoleucine | -0.2526299 | 0.00967303 | 0.62633197 | Advice | Not Significant | Not Significant |
| Isoleucine | -0.2787043 | 0.00498711 | 0.64787096 | Inulin | Not Significant | Not Significant |
| Leucine | 0.24876003 | 0.01088514 | 0.64874809 | Placebo | Not Significant | Not Significant |
| Leucine | 0.29033857 | 0.00279178 | 0.71982096 | Advice | Not Significant | Not Significant |
| Leucine | 0.32177026 | 0.00109648 | 0.74798141 | Inulin | Not Significant | Not Significant |
| Lysine | -0.3242085 | 0.00078651 | 0.84551232 | Placebo | Not Significant | Not Significant |
| Lysine | -0.2531472 | 0.00952036 | 0.62761432 | Advice | Not Significant | Not Significant |
| Lysine | -0.2020804 | 0.04377391 | 0.4697524 | Inulin | Not Significant | Not Significant |
| Met-SO | -0.3376646 | 0.00045585 | 0.88060473 | Placebo | Not Significant | Not Significant |
| Met-SO | -0.2797274 | 0.00402957 | 0.69351333 | Advice | Not Significant | Not Significant |
| Met-SO | -0.399794 | 3.77E-05 | 0.92935413 | Inulin | Not Significant | Not Significant |
| Methionine | 0.62112215 | 2.00E-12 | 1.61984146 | Placebo | Significant | Significant |
| Methionine | 0.67915595 | 2.29E-15 | 1.68379521 | Advice | Significant | Significant |
| Methionine | 0.71569762 | 5.88E-17 | 1.66369793 | Inulin | Significant | Significant |
| Nitro-Tyr | -0.3690376 | 0.00011575 | 0.96242316 | Placebo | Not Significant | Not Significant |
| Nitro-Tyr | -0.4132246 | 1.30E-05 | 1.02448564 | Advice | Not Significant | Significant |
| Nitro-Tyr | -0.4056485 | 2.83E-05 | 0.94296328 | Inulin | Not Significant | Not Significant |
| Ornitine | 0.72405636 | 3.79E-18 | 1.8882864 | Placebo | Significant | Significant |
| Ornitine | 0.59973263 | 1.73E-11 | 1.4868852 | Advice | Significant | Significant |
| Ornitine | 0.74587382 | 5.44E-19 | 1.733845 | Inulin | Significant | Significant |
| PEA | 0.09407691 | 0.34215629 | 0.24534575 | Placebo | Not Significant | Not Significant |
| PEA | -0.0382504 | 0.69986293 | 0.09483215 | Advice | Not Significant | Not Significant |
| PEA | 0.13009232 | 0.19703079 | 0.30241028 | Inulin | Not Significant | Not Significant |
| Phenylalanine | 0.22339772 | 0.02263298 | 0.58260502 | Placebo | Not Significant | Not Significant |
| Phenylalanine | 0.35772912 | 0.00019285 | 0.88689878 | Advice | Not Significant | Not Significant |
| Phenylalanine | 0.30804076 | 0.00182167 | 0.71606607 | Inulin | Not Significant | Not Significant |
| Proline | 0.03704287 | 0.70890504 | 0.09660511 | Placebo | Not Significant | Not Significant |
| Proline | -0.1179953 | 0.23288895 | 0.29253956 | Advice | Not Significant | Not Significant |
| Proline | -0.0613972 | 0.54396644 | 0.14272288 | Inulin | Not Significant | Not Significant |
| Putrescine | 0.30949214 | 0.00138893 | 0.80713303 | Placebo | Not Significant | Not Significant |
| Putrescine | 0.39190566 | 3.89E-05 | 0.97163084 | Advice | Not Significant | Not Significant |
| Putrescine | 0.60054947 | 3.98E-11 | 1.39602661 | Inulin | Significant | Significant |
| SDMA | 0.4948435 | 9.31E-08 | 1.29051591 | Placebo | Not Significant | Significant |
| SDMA | 0.53137542 | 6.49E-09 | 1.3174108 | Advice | Significant | Significant |
| SDMA | 0.62340332 | 4.29E-12 | 1.44915225 | Inulin | Significant | Significant |
| Serotonin | -0.2401349 | 0.01407485 | 0.62625436 | Placebo | Not Significant | Not Significant |
| Serotonin | -0.3991203 | 2.71E-05 | 0.98951773 | Advice | Not Significant | Not Significant |
| Serotonin | -0.2953948 | 0.00284793 | 0.68666956 | Inulin | Not Significant | Not Significant |
| Spermidine | -0.4450295 | 2.21E-06 | 1.16060461 | Placebo | Not Significant | Significant |
| Spermidine | -0.4675559 | 5.61E-07 | 1.15918643 | Advice | Not Significant | Significant |
| Spermidine | -0.347336 | 0.00039923 | 0.80741113 | Inulin | Not Significant | Not Significant |
| Spermine | 0.38670728 | 5.02E-05 | 1.00850449 | Placebo | Not Significant | Significant |
| Spermine | 0.47554312 | 3.37E-07 | 1.17898876 | Advice | Not Significant | Significant |
| Spermine | 0.53170102 | 1.25E-08 | 1.23598273 | Inulin | Significant | Significant |
| t4-OH-Pro | 0.430913 | 4.96E-06 | 1.12378981 | Placebo | Not Significant | Significant |
| t4-OH-Pro | 0.47760605 | 2.95E-07 | 1.18410326 | Advice | Not Significant | Significant |
| t4-OH-Pro | 0.51612562 | 3.88E-08 | 1.19977643 | Inulin | Significant | Significant |
| Taurine | -0.0115876 | 0.90706136 | 0.03021949 | Placebo | Not Significant | Not Significant |
| Taurine | -0.1340341 | 0.17494206 | 0.3323036 | Advice | Not Significant | Not Significant |
| Taurine | -0.2547246 | 0.01054105 | 0.59212819 | Inulin | Not Significant | Not Significant |
| Threonine | 0.14333866 | 0.14660917 | 0.37381681 | Placebo | Not Significant | Not Significant |
| Threonine | -0.0766452 | 0.43933176 | 0.19002233 | Advice | Not Significant | Not Significant |
| Threonine | 0.04497057 | 0.65684257 | 0.10453779 | Inulin | Not Significant | Not Significant |
| Tryptophan | 0.40365926 | 2.15E-05 | 1.05271404 | Placebo | Not Significant | Significant |
| Tryptophan | 0.40083946 | 2.48E-05 | 0.99377995 | Advice | Not Significant | Not Significant |
| Tryptophan | 0.31817109 | 0.00125545 | 0.73961484 | Inulin | Not Significant | Not Significant |
| Tyrosine | 0.40601065 | 1.90E-05 | 1.05884628 | Placebo | Not Significant | Significant |
| Tyrosine | 0.4729212 | 3.99E-07 | 1.17248836 | Advice | Not Significant | Significant |
| Tyrosine | 0.53529385 | 9.58E-09 | 1.24433454 | Inulin | Significant | Significant |
| Valine | -0.2798541 | 0.00401229 | 0.72983914 | Placebo | Not Significant | Not Significant |
| Valine | -0.1961405 | 0.04598829 | 0.48628069 | Advice | Not Significant | Not Significant |
| Valine | -0.3052414 | 0.00201448 | 0.70955862 | Inulin | Not Significant | Not Significant |

Ac-Orn, acetylornithine; ADMA, asymmetric dimethylarginine; alpha-AAA, alpha-aminoadipic acid; c4-OH-Pro, cis-4-hydroxyproline; DOPA, dihydroxyphenylalanine; Met-SO, methionine-sulfoxide; Nitro-Tyr, nitrotyrosine; PEA, phenylethylamine; SDMA, symmetric dimethylarginine; t4-OH-Pro, trans-4-hydroxyproline.

**Supplementary Table S5** Between-group analysis of amino acid and biogenic amine concentration differences. Results of one-way ANOVA comparing amino acid and biogenic amine concentrations across groups, including all compounds analyzed to evaluate group differences. Compounds with more than 20% missing values were excluded from the analysis.

| **Amino acids and biogenic amines** | **ANOVA P-value*** |
| --- | --- |
| Ala | 0.904881559 |
| Arg | 0.244580137 |
| Asp | 0.594362608 |
| Cit | 0.294137639 |
| Gln | 0.422026607 |
| Glu | 0.880304515 |
| Gly | 0.819239927 |
| His | 0.708409439 |
| Ile | 0.930108688 |
| Leu | 0.90034084 |
| Lys | 0.904213491 |
| Met | 0.485697209 |
| Orn | 0.587044475 |
| Phe | 0.821484912 |
| Pro | 0.735166714 |
| Thr | 0.419296799 |
| Trp | 0.646101416 |
| Tyr | 0.821243159 |
| Val | 0.361833521 |
| Ac-Orn | 0.953447876 |
| ADMA | 0.884135801 |
| Creatinine | 0.448379717 |
| Dopamine | 0.636887998 |
| Met-SO | 0.874327356 |
| Putrescine | 0.009862882 |
| SDMA | 0.693685445 |
| Spermine | 0.12060729 |
| t4-OH-Pro | 0.315772317 |
| Taurine | 0.509521726 |

*one-way analysis of variance P-value.

Ac-Orn, acetylornithine; ADMA, asymmetric dimethylarginine; Ala, alanine; Arg, arginine; Asp, aspartate; Cit, citrulline; Gln, glutamine; Glu, glutamate; Gly, glycine; His, histidine; Ile, isoleucine; Leu, leucine; Lys, lysine; Met, methionine; Met-SO, methionine-sulfoxide; Orn, ornithine; Phe, phenylalanine; Pro, proline; SDMA, symmetric dimethylarginine; Thr, threonine; Trp, tryptophan; Tyr, tyrosine; t4-OH-Pro, trans-4-hydroxyproline; Val, valine.
