# Supplementary material for: Inulin supplementation modulates gut microbiota derived metabolites related to brain function in children with obesity
Source: Sci Rep. 2025 Oct 7;15:34843. doi: 10.1038/s41598-025-21079-2 (PMC12504693; doi:10.1038/s41598-025-21079-2)
Supplement: Supplementary file 2 — Supplementary Material 2 [file 41598_2025_21079_MOESM2_ESM.docx]

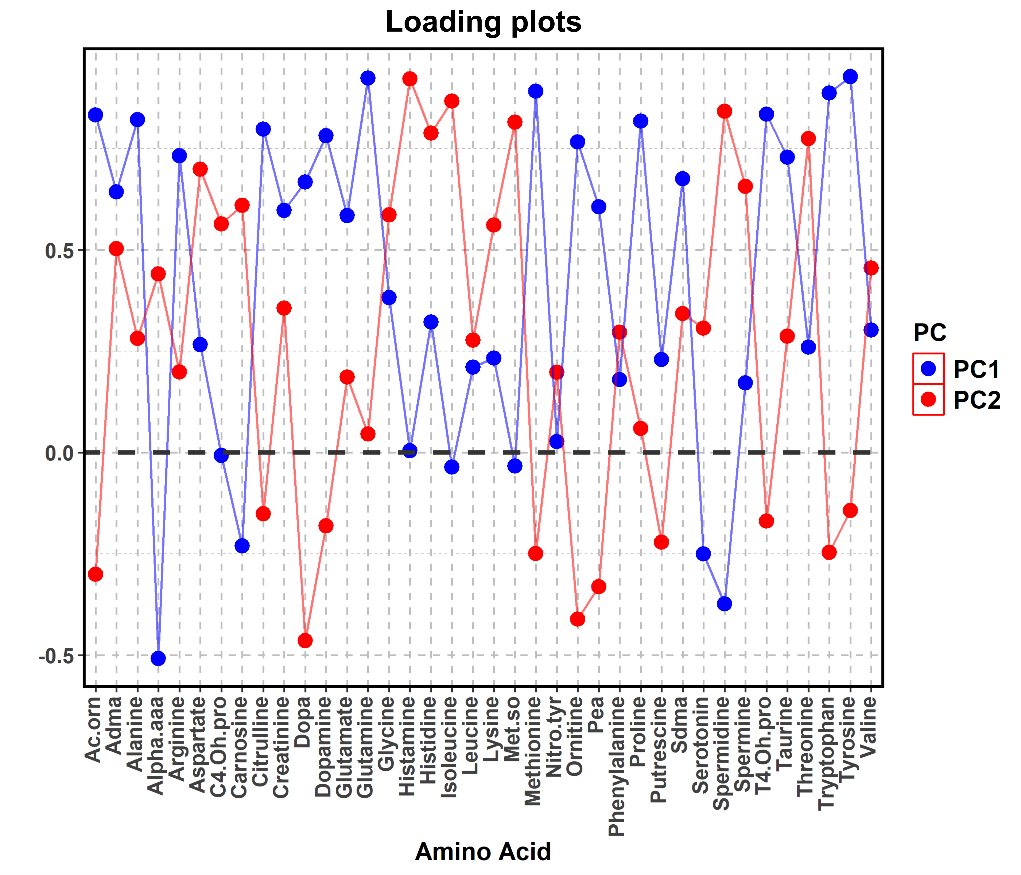


**Supplementary Fig. S1** Loading plots of individual amino acids and biogenic amines on the first two principal components (PC1 and PC2) from baseline (month 0) to six months (month 6) across the placebo, inulin, and dietary fiber advice groups.

Ac.Orn, acetylornithine; Adma, asymmetric dimethylarginine; Alpha.aaa, alpha-aminoadipic acid; C4-Oh-pro, cis-4-hydroxyproline; Dopa, dihydroxyphenylalanine; Met-so, methionine-sulfoxide; Nitro-tyr, nitrotyrosine; Pea, phenylethylamine; Sdma, symmetric dimethylarginine; T4-Oh-pro, trans-4-hydroxyproline.


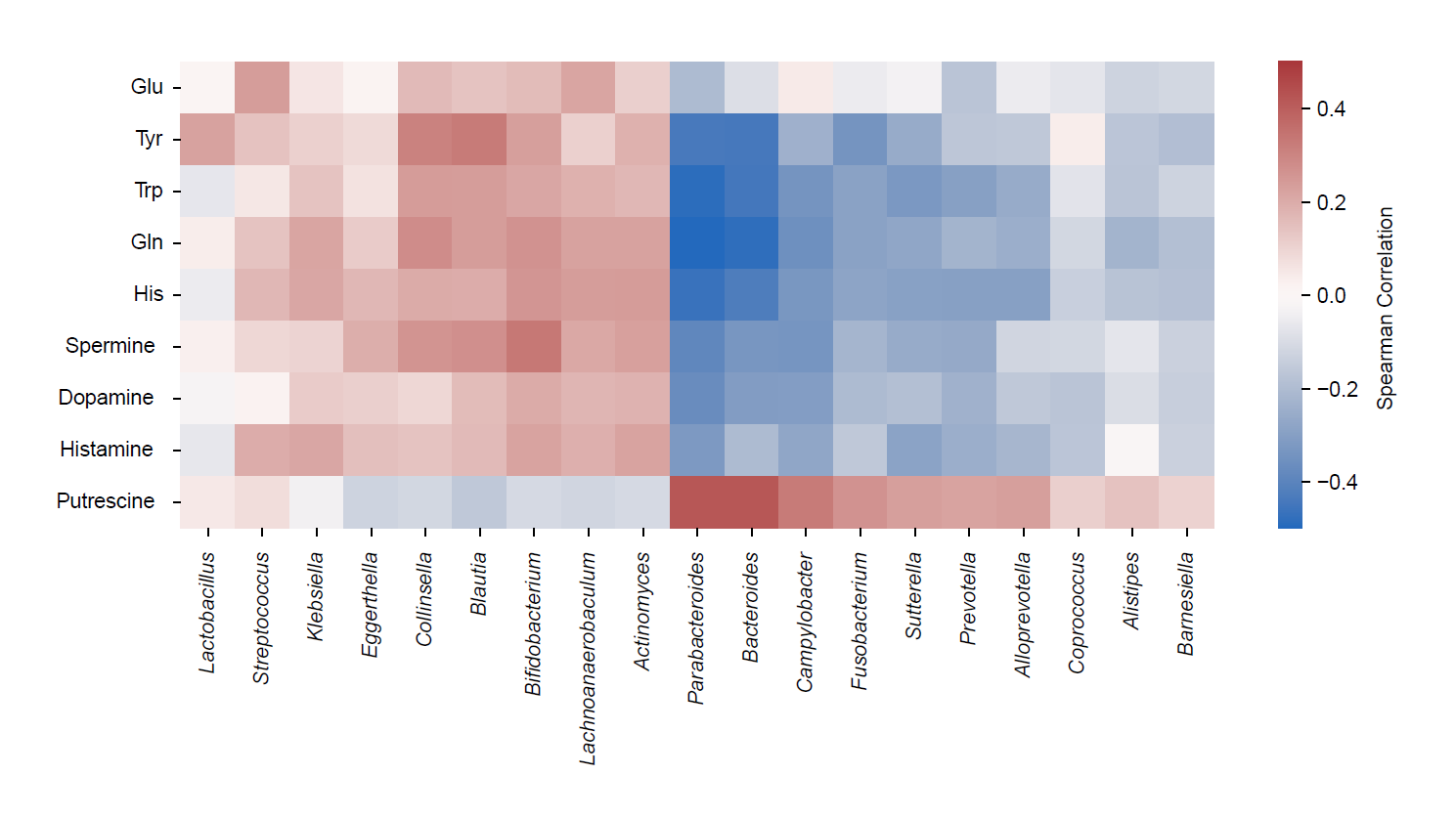


**Supplementary Fig. S2** Relationships of GBA-related amino acids and biogenic amines with gut microbiota at baseline by heatmap, analyzed by Spearman’s correlation coefficient with Benjamini-Hochberg correction with FDR = 0.05.

GBA, gut-brain axis; Gln, glutamine; Glu, glutamate; His, histidine; Trp, tryptophan; Tyr, tyrosine.


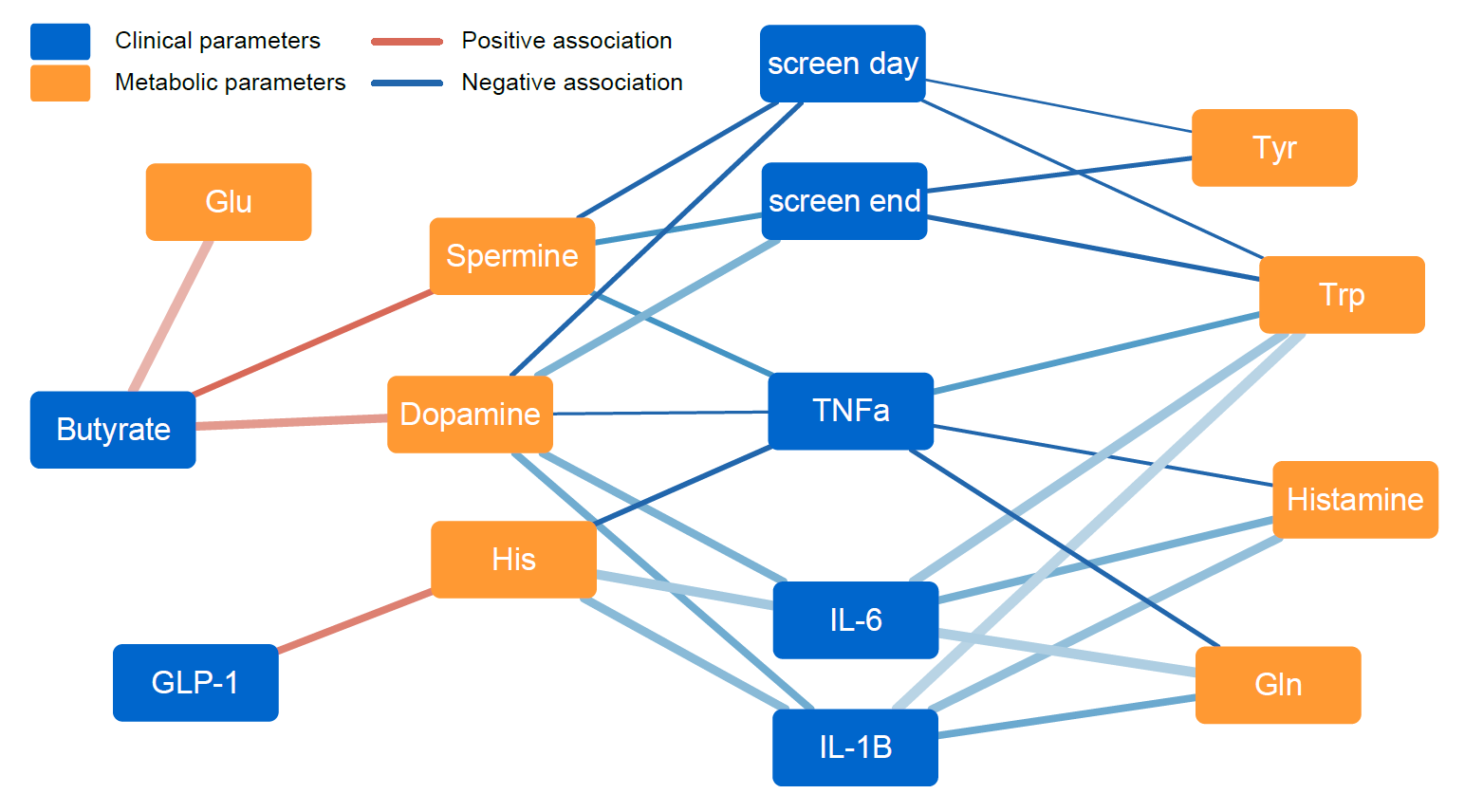


**Supplementary Fig. S3** Relationships of GBA-related amino acids and biogenic amines with physical activity and biochemical parameters at baseline by correlation network, analyzed by Spearman’s correlation coefficient with Benjamini-Hochberg correction with FDR = 0.05.

GBA, gut-brain axis; Gln, glutamine; Glu, glutamate; GLP-1, glucagon-like peptide 1; His, histidine; IL-1β, interleukin-1β; IL-6, interleukin-6; screen day, screen time on weekdays; screen end, screen time on weekends, TNFa, tumor necrosis factor-α; Trp, tryptophan; Tyr, tyrosine.


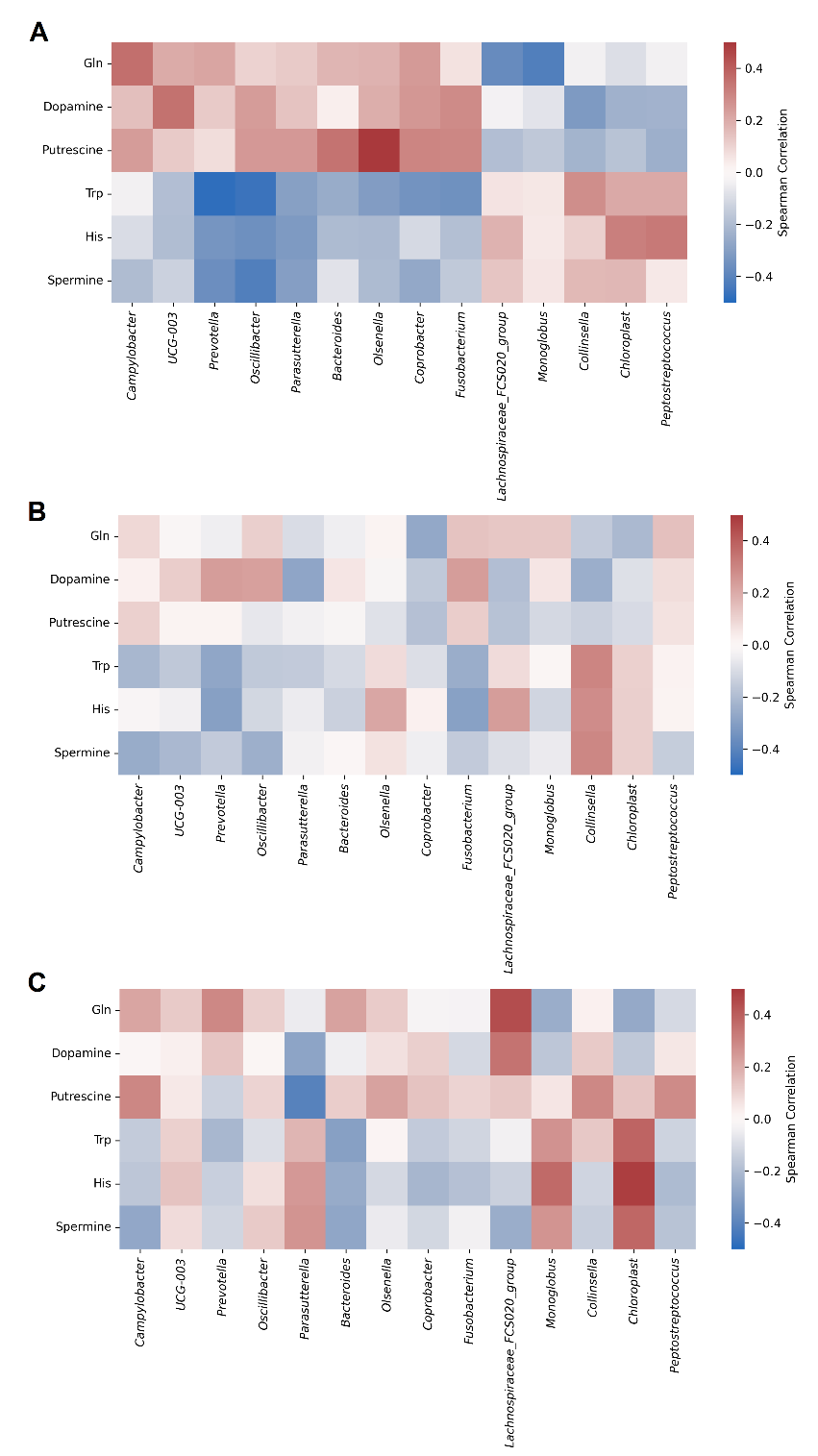


**Supplementary Fig. S4** Relationships of changes in GBA-related amino acids and biogenic amines with changes in gut microbiota after intervention by heatmap, analyzed by Spearman’s correlation coefficient with Benjamini-Hochberg correction with FDR = 0.05. (A) Inulin group, (B) Placebo group, and (C) Dietary fiber advice group.

GBA, gut-brain axis; Gln, glutamine; His, histidine; Trp, tryptophan.
